# Potential costs of heterospecific sexual interactions in golden orbweb spiders (*Nephila* spp.)

**DOI:** 10.1038/srep36908

**Published:** 2016-11-15

**Authors:** Shakira G. Quiñones-Lebrón, Simona Kralj-Fišer, Matjaž Gregorič, Tjaša Lokovšek, Klemen Čandek, Charles R. Haddad, Matjaž Kuntner

**Affiliations:** 1 Evolutionary Zoology Laboratory, Biological Institute ZRC SAZU, Ljubljana, Slovenia; 2 Department of Zoology & Entomology, University of the Free State, Bloemfontein, South Africa; 3 Department of Entomology, National Museum of Natural History, Smithsonian Institution, Washington, D.C., USA; 4 Centre for Behavioural Ecology & Evolution (CBEE), College of Life Sciences, Hubei University, Wuhan, China

## Abstract

Though not uncommon in other animals, heterospecific mating is rarely reported in arachnids. We investigated sexual interactions among four closely related and syntopical African golden orbweb spiders, *Nephila inaurata*, *N. fenestrata*, *N. komaci*, and *N. senegalensis*. In two South African localities, female webs were often inhabited by heterospecific males that sometimes outnumbered conspecifics. Species association of males with females was random in nature. In subsequent laboratory choice experiments, *N. inaurata* males chose heterospecific females in 30% of trials. We also observed natural mating interactions between *N. inaurata* males and *N. komaci* females, and between *N. komaci* males and *N. inaurata* females in laboratory experiments. While heterospecific mating in the laboratory never produced offspring, conspecific mating did. We discuss potential ecological and evolutionary consequences of heterospecific mating interactions in *Nephila* that may be particularly costly to the rarer species.

Closely related species with overlapping ranges that compete for resources are often subjected to ecological research, e.g. in studies of species distribution^1^ and competition for resources^2^,^3^. Less attention has been given to species interacting in a mating context, beyond the obvious cases of hybridization. Sexual encounters between species may be costly and non-adaptive, but are not uncommon, with 167 species spanning the animal kingdom reported to engage in sexual interactions with heterospecifics^4^. When heterospecific sexual interactions affect the fitness of individuals of either species, theory predicts the occurrence of reproductive interference^5^. Reproductive interference typically results from incomplete species recognition, reinforced by strong male-male competition, resulting in indiscriminate male mating attempts and relaxed female choice^5^. While reproductive interference may be common^6^, the effects on population dynamics and community structure remain unexplored.

In arachnids, heterospecific sexual interactions are confined to spider mites^7^,^8^, and two anecdotal accounts exist of laboratory-staged mating trials in cursorial spiders, Ctenidae^9^ and Lycosidae^10^, but have so far not been reported in orbweb spiders. Heterospecific sexual interactions are expected to be rare in species where sexual cannibalism is prevalent, since, in theory, cannibalism in the context of female choice would allow females to avert such mistakes. One may thus expect that spiders, particularly sexually dimorphic taxa known for sexual cannibalism^11^, would not engage in heterospecific matings.

However, our field discovery and subsequent laboratory studies, surprisingly, suggest otherwise. We investigated field sex ratios in, and species interactions between, four golden orbweb spiders (genus *Nephila*) co-occurring in an area in north-eastern KwaZulu-Natal, South Africa: *Nephila inaurata* (Walckenaer, 1841), *N. fenestrata* (Thorell, 1859), *N. komaci* Kuntner & Coddington, 2009, and *N. senegalensis* (Walckenaer, 1841). Their relative phylogenetic proximity^12^, their co-occurrence, and similar life histories suggest interspecific competition for niche and mating opportunities. In particular, *N. inaurata* and *N. komaci* are ecologically indistinguishable where they co-habit, but *N. inaurata* is widespread and locally abundant^13^ whereas *N. komaci* is severely limited in range and locally rare^14^. We report on the occurrence of heterospecific interactions among the four *Nephila* species in the field, and of mating between *N. komaci* and *N. inaurata.* These interactions are surprising given that sexual cannibalism is common in *Nephila*^12^, and could play an important role in determining the community structure of *Nephila*, possibly through reproductive interference.

## Results

**Figure 1:**
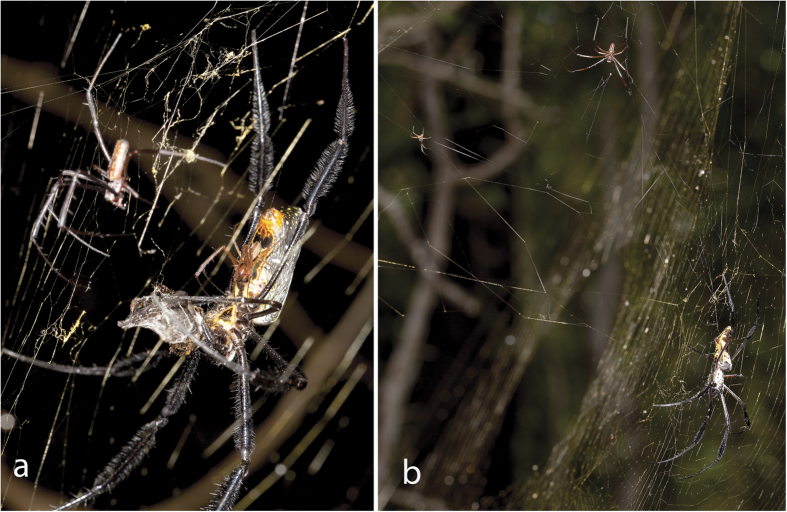
Interspecific interactions in *Nephila*. (**a**) feeding female *N. fenestrata* with a conspecific male mating, and a *N. inaurata* male (left, large) within her web in iSimangaliso NP. (**b**) female *N. komaci* (lower, right), with a conspecific (left, small) and a heterospecific male (*N. inaurata*, top, large) within her web in Ndumo Game Reserve.

We observed a total of 127 haphazardly encountered females of the four *Nephila* species in the two sites in KwaZulu-Natal, South Africa. Most webs of subadult and adult females were occupied by at least one male. Of these, heterospecific males were present on 50%, 20–60%, and 66.67% of *N. fenestrata*, *N. inaurata*, and *N. komaci* webs, respectively (Fig. 1; Table 1).

In iSimangaliso Wetland Park, we observed 29 *N. fenestrata* and 32 *N. inaurata* females. On the webs of both species we observed conspecific as well as heterospecific males. Sixty-two percent of *N. fenestrata* females (n = 18) had at least one male cohabiting in the web (2.5 ± 1.67, n = 44 males). Of the recovered males (n = 35), 31% (n = 11) were heterospecific. We observed a total of 24 males in 10 of the observed *N. inaurata* webs, including juvenile webs (2.56 ± 1.71); of the collected males (n = 17), 10 were heterospecific (59%).

In Ndumo Game Reserve, 83% (10 of 12 webs) of *N. inaurata* webs were inhabited by at least one male (1.40 ± 0.66; n = 14 males), and two of the collected males (n = 9) were heterospecific (22%). We observed 37 webs of female *N. komaci*, of which 65% (n = 24) were inhabited by at least one male (1.50 ± 0.82, n = 36 males), these being mostly heterospecific (65%, n = 17 of 26 collected males). We observed 17 *N. senegalensis* webs. Only nine males were found on *N. senegalensis* webs (1.13 ± 0.33), of which four were conspecifics; the other five males were lost and could not be identified. We observed two copulations between *N. inaurata* males and *N. komaci* females.

Male association with webs was random between *N. inaurata* and *N. fenestrata* in iSimangaliso (Fisher’s exact test, n = 52, p = 0.544), and not random among the three species in Ndumo (Fisher’s exact test, n = 39, p < 0.001); there, *N. komaci* webs were occupied predominantly by heterospecific males (65.38%, n = 17 heterospecific males; 34.62%, n = 9 conspecific males).

**Figure 2:**
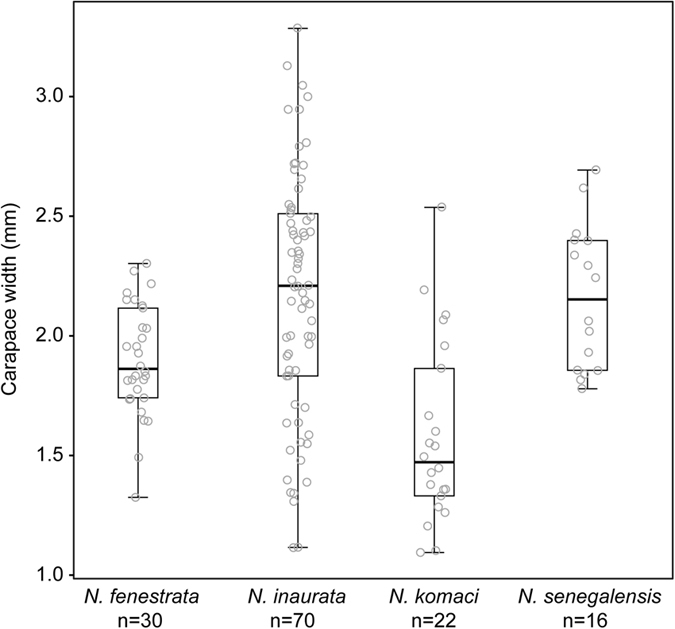
Comparisons of male carapace width for the four *Nephila* species (raw data and box plots). *N. inaurata* males were significantly larger than *N. fenestrata* and *N. komaci. N. senegalensis* males were also significantly larger than *N. komaci*.

Male sizes varied among (ANOVA: *F*_134,3_ = 12.482, p < 0.001) and within species (Fig. 2). Post hoc comparisons using Tukey HSD revealed that *N. inaurata* males were larger than *N. fenestrata* males (p-adjust = 0.02, n = 100), while *N. komaci* males were smaller than both *N. inaurata* (p-adjust < 0.001, n = 92) and *N. senegalensis* males (p-adjust < 0.001, n = 38).

**Figure 3:**
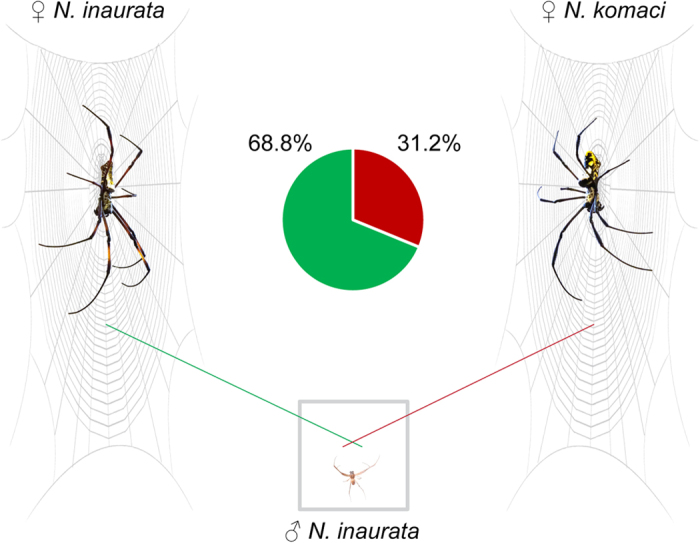
Laboratory choice experiment. *Nephila inaurata* males were given the choice between a *N. inaurata* female and a *N. komaci* female (n = 16).

In mate choice experiments (Fig. 3), *N. inaurata* males chose conspecific versus heterospecific females in 11/16 (68.75%), versus in 5/16 trials (31.25%), respectively. Conspecific and heterospecific mate choice occurred randomly (binomial test: p = 0.21, n = 16). In these trials, one male copulated with a conspecific female. We observed courtship, but no copulation, in additional four conspecific couples and two heterospecific couples. We observed female aggression in four cases, three of which occurred in conspecific couples.

In mating trials between *N. komaci* males and *N. inaurata* females, all males (n = 9) courted females, and of these, eight males (88.89%) mated using both palps. We monitored all heterospecifically mated females, of which only two produced egg-sacs, but these were unviable.

## Discussion

We report on the first heterospecific sexual interactions among orbweb spiders. Our field and laboratory results suggest that heterospecific coexistence in webs, and even mating interactions, are common in *Nephila*. We predominantly detected random species association of males with females in nature, and found no preference for conspecific females by *N. inaurata* males in the laboratory trials. Our results suggest that heterospecific interactions of *Nephila* males may involve several mistakes in locating, courting to, and mating with a “correct” (conspecific) female. As explained below, the phenomenon might be a result of incomplete species recognition reinforced by male – male competition and male biased sex ratios.

The high proportions of *Nephila* webs occupied by heterospecific males in nature (20–60%) may be a consequence of incomplete species recognition, where males randomly occupy heterospecific and conspecific webs of congeners. Random mate choice was also confirmed in laboratory experiments, however, *N. inaurata* males chose a conspecific female in 70% of trials. On the other hand, in the absence of a choice between a con- and a heterospecific female, *N. komaci* males always engaged in mating interactions with heterospecific females (*N. inaurata*). These results imply that *Nephila* males are in fact able, to some degree, to identify conspecifics on webs, but when on heterospecific webs, they indiscriminately engage in mating interactions.

*Nephila* spiders are extremely sexually size dimorphic, and occur in male biased sex ratios^15^,^16^. Females commonly co-habit with multiple males, which compete for mating opportunities. In direct male – male competition larger males usually outcompete smaller rivals^17^,^18^,^19^,^20^. On average among the species studied here, *N. komaci* males are the smallest and are thus expected to be outcompeted, in heterospecific interactions, by larger *N. inaurata* or *N. senegalensis*. Indeed, our field observations found that most males in *N. komaci* webs (~65%) were heterospecific. Additionally, local variation in species composition and phenology, relative abundances, and species-specific sex ratios likely affect the proportion of heterospecific males. For example, the proportion of heterospecific males in *N. inaurata* webs greatly differ between the two locations in our study. In iSimangaliso Wetland Park, where *N. inaurata* co-occurs with *N. fenestrata,* 60% of *N. inaurata* webs harbored heterospecific males. On the other hand, in Ndumo Game Reserve, where *N. inaurata* co-occurs with *N. komaci* and *N. senegalensis,* only 20% of *N. inaurata* webs harbored heterospecific males.

Heterospecific sexual interactions could interfere with reproduction outputs of sympatric species. If so, occupying a heterospecific female web could affect male, perhaps even female fitness. Several risks exist for males. First, strong male-male competition in *Nephila* usually results in larger males outcompeting smaller ones^17^,^18^,^19^,^20^, and thus the syntopic species with smaller males is likely at a disadvantage. For example, *N. komaci* males are on average smaller than the other two co-occurring species, which implies that interspecies male competition could lower their reproductive success simply due to an increased number of competing, larger males. This may explain the rarity of *N. komaci* in nature. In addition to male competition, males also risk being cannibalized by females, as is common in *Nephila*^11^,^21^. Finally, if at least some sperm gets transferred at copulations with heterospecific females, males risk becoming sperm depleted because adult *Nephila* males are incapable of recharging their palps with sperm^22^,^23^.

For females, heterospecific males are a threat to their own reproductive success. Failure in species recognition poses a cost, as females need to balance between being choosy (by means of sexual cannibalism) on the one hand, and accepting enough males in order to successfully reproduce on the other. Because of female gigantism, *Nephila* females are expected to be sperm depleted, and at least in laboratory conditions, they need to mate more than twice before they produce a viable eggsac^24^,^25^. Moreover, none of the females that in our trials mated with both a conspecific and a heterospecific were able to produce viable egg sacs, while those that only mated with conspecifics did. While this evidence is preliminary, it suggests that heterospecific mating negatively affects female fitness. A similar phenomenon has been observed between two closely related *Panonychus* spider mites, where females that had mated with a conspecific after having mated with a heterospecific, did not produce female offspring^7^.

The observed heterospecific sexual interactions present an opportunity to explore a theoretical framework in which reproductive interference would play a key role in the population dynamics of each of the represented species, and interspecific competition. It is reasonable to believe that the presence of males from the dominant species *– N. inaurata* – on webs of the globally (*N. komaci*) or locally rare species (*N. senegalensis*) could decrease their reproductive rates. However, together with this, other factors such as climatic variation, individual species’ biology, inter- and intraspecific competition, annual fluctuations of natural enemies, amongst others, may all contribute to the considerable annual population fluctuations of each of these species in Maputaland. In the case of reproductive interference, the locally dominant species could also suffer from sperm depletion if too many males are failing to arrive at the correct/conspecific females, therefore also suffering a reduction in reproductive output. Such dynamics have been hypothesized to maintain the coexistence of ecologically identical species^26^.

A model of coexistence through reproductive interference^26^ applies to species using ephemeral patchy resources, which does not apply to our focal species. More inclusive population and ecological models may illuminate implications of reproductive interference for rare species like *N. komaci*, living in habitats of continuous resources (see Supplementary material 1). Coexistence could be possible if the rarer species has a greater impact on the reproductive output of the common species. On the contrary, if the asymmetry of the interference favors the dominant species, the rare species (*N. komaci*) would potentially become locally extinct. Since in most of the *Nephila* range, single species dominate, the here investigated coexistence of the four species is secondary, and may not be ecologically stable.

## Methods

### Specimen collection

We observed and collected specimens of *N. fenestrata* and *N. inaurata* in iSimangaliso Wetland Park (S28.15895, E32.28897), and of *N. inaurata*, *N. komaci* and *N. senegalensis* in Ndumo Game Reserve (S26.54217, E32.15957) in north-eastern KwaZulu-Natal, South Africa (southern Maputaland) for two weeks in February 2015. We counted all males present on each encountered *Nephila* web and the female’s ontogenetic stage. For subsequent body size measurements, we photographed each spider using a Canon Eos 7D camera equipped with a Canon 50 mm macro lens. For all photography of spiders, we used a standardized fixed focal length. The photos were used to calculate carapace width as a measure of male size, for 30 *N. fenestrata*, 70 *N. inaurata*, 22 *N. komaci*, and 16 *N. senegalensis* males (Fig. 2). After the field survey, we transferred live specimens of *N. inaurata* and *N. komaci* to the laboratory for additional experiments.

### Spider husbandry

Adult females were kept in separate (60 × 60 × 12 cm) acrylic glass frames in the laboratory where light/dark cycles and temperature were controlled (LD 12:12 h, T = 20 °C). Males and small juveniles were kept in 200 mL plastic cups under the same conditions. All spiders were water-sprayed daily and fed twice a week. Large females were fed with 4–5 flies or one mealworm, while juveniles and males were fed with one blowfly or several fruit flies.

### Mate choice experiments

We tested *N. inaurata* males for discriminating between a conspecific and a heterospecific female (n = 22). We placed a frame inhabited by female *N. inaurata* parallel to a frame with *N. komaci* female such that both frames shared a side opening (Fig. 3). We pulled a silk thread from each female web and attached it to a polystyrene platform placed in-between the two frames. We positioned the two silk threads in such a way that they crossed each other to allow the male to touch both threads simultaneously. We then gently placed a *N. inaurata* male on the polystyrene platform and positioned him to touch both threads. During a 60 min timeframe, we observed male choice for either of the two webs, the occurrence of courting, female aggression, and copulation. We determined choice when the male positioned himself on one of the females’ webs (n = 16, Fig. 3). We excluded those trials where males did not start climbing any thread or did not climb onto either web (n = 6). All females were mated with conspecifics prior to the choice experiments to control for their mating status.

### Heterospecific mating trials

To confirm heterospecific mating, we performed mating trials between *N. komaci* males and *N. inaurata* females (n = 9). We placed a male on the attachment threads of the web of a heterospecific female for up to five hours to observe the occurrence of courtship and mating attempts. The female was given prey at the beginning of the mating trial in order to avoid sexual cannibalism due to hunger. We determined mating interaction when we observed that a male palpal insertion into female genital opening resulted in hematodocha swelling, which usually indicates sperm transfer^25^. However, actual sperm transfer to female spermathecae could not be confirmed. To test for hybridization, we collected egg-sacs produced by females that mated with heterospecific males. Egg-sacs were kept at 20 °C and sprayed daily with water for two months. Egg-sacs that failed to hatch within two months were considered unviable.

### Statistical analyses

We tested whether male occupancy on female webs was random or biased towards conspecific/heterospecific females using Fisher’s Exact Test. Male mate choice was tested for randomness using a binomial test. We compared male size (i.e. carapace width) among species using one-way analysis of variance. All statistical tests were performed in R (R Development Core Team 2009).

## Additional Information

**How to cite this article**: Quiñones-Lebrón, S. G. *et al.* Potential costs of heterospecific sexual interactions in golden orbweb spiders (*Nephila* spp.). *Sci. Rep.* **6**, 36908; doi: 10.1038/srep36908 (2016).

**Publisher’s note:** Springer Nature remains neutral with regard to jurisdictional claims in published maps and institutional affiliations.

## Supplementary Material

Supplementary Information

## Figures and Tables

**Table 1:** Summary of surveyed *Nephila* webs at two South African locations.

Species	Total webs with males	Webs with con. males	Webs with het. males	Total males	Lost males	Heterospecific males
*N. fenestrata* iSi, n = 29	18 (62%)	13 (72%)	9 (50%)	44	9	11 (31%)
*N. inaurata* iSi, n = 32	10 (31%)	7 (70%)	6 (60%)	24	7	10 (59%)
*N. inaurata* Ndumo, n = 12	10 (83%)	7 (70%)	2 (20%)	14	5	2 (22%)
*N. komaci* Ndumo, n = 37	24 (65%)	8 (33%)	16 (67%)	36	10	17 (65%)
*N. senegalensis* Ndumo, n = 17	8 (41%)	3 (38%)	0 (0%)	9	5	0 (0%)

Note that several males could not be identified because they were lost at site. Legend: iSi = iSimangaliso Wetland Park; Ndumo = Ndumo Game Reserve.

## References

[b1] Thum R. A. Reproductive interference, priority effects and the maintenance of parapatry in *Skistodiaptomus* copepods. Oikos 116, 759–768 (2007).

[b2] Harper J. L., Clatworthy J. N., Mcnaughton I. H. & Sagar G. R. The evolution and ecology of closely related species living in the same area. Evolution 15, 209–227 (1961).

[b3] Valiente-Banuet A. & Verdú M. Temporal shifts from facilitation to competition occur between closely related taxa. J. Ecol. 96, 489–494 (2008).

[b4] Gröning J. & Hochkirch A. Reproductive interference between animal species. Q. Rev. Biol. 83, 257–282 (2008). 18792662 10.1086/590510

[b5] Burdfield-Steel E. R. & Shuker D. M. Reproductive interference. Curr. Biol. 21, R450–R451 (2011). 21683894 10.1016/j.cub.2011.03.063

[b6] Kyogoku D. Reproductive interference: ecological and evolutionary consequences of interspecific promiscuity. Popul. Ecol. 57, 253–260 (2015).

[b7] Takafuji A., Kuno E. & Fujimoto H. Reproductive interference and its consequences for the competitive interactions between two closely related *Panonychus* spider mites. Exp. Appl. Acarol. 21, 379–391 (1997).

[b8] Ben-David T., Gerson U. & Morin S. Asymmetric reproductive interference between two closely related spider mites: *Tetranychus urticaev* and *T. turkestani* (Acari: Tetranychidae). Exp. Appl. Acarol. 48, 213–227 (2009). 19160063 10.1007/s10493-008-9228-9

[b9] Schmitt A. Conjectures on the origins and functions of a bridal veil spun by the males of *Cupiennius coccineus* (Araneae, Ctenidae). J. Arachnol. 20, 67–68 (1992).

[b10] Kronestedt T. A case of heterospecific mating in wolf spiders (Araneae, Lycosidae). J. Arachnol. 22, 84–86 (1994).

[b11] Elgar M. A. Sexual cannibalism, size dimorphism, and courtship behavior in orb-weaving spiders (Araneidae). Evolution 45, 444–448 (1991). 10.1111/j.1558-5646.1991.tb04419.x 28567867

[b12] Kuntner M., Arnedo M. A., Trontelj P., Lokovšek T. & Agnarsson I. A molecular phylogeny of nephilid spiders: Evolutionary history of a model lineage. Mol. Phylogenet. Evol. 69, 961–979 (2013). 23811436 10.1016/j.ympev.2013.06.008

[b13] Kuntner M. & Agnarsson I. Phylogeography of a successful aerial disperser: the golden orb spider *Nephila* on Indian Ocean islands. BMC Evol. Biol. 11, 119 (2011). 21554687 10.1186/1471-2148-11-119 PMC3098804

[b14] Kuntner M. & Coddington J. A. Discovery of the largest orbweaving spider species: The evolution of gigantism in *Nephila*. PLoS One 4, e7516 (2009). 19844575 10.1371/journal.pone.0007516 PMC2760137

[b15] Kuntner M. & Cheng R. C. In Evolutionary biology: Convergent evolution, evolution of complex traits, concepts and methods (ed. Pontarotti P.) 121–133 (Springer International Publishing, 2016).

[b16] Kuntner M. & Elgar M. A. Evolution and maintenance of sexual size dimorphism: Aligning phylogenetic and experimental evidence. Front. Ecol. Evol. 2, 1–8 (2014).

[b17] Constant N., Valbuena D. & Rittschof C. C. Male contest investment changes with male body size but not female quality in the spider *Nephila clavipes*. Behav. Processes 87, 218–223 (2011). 21513777 10.1016/j.beproc.2011.04.003

[b18] Elgar M. A., De Crespigny F. E. C. & Ramamurthy S. Male copulation behaviour and the risk of sperm competition. Anim. Behav. 66, 211–216 (2003).

[b19] Fromhage L. & Schneider J. M. Safer sex with feeding females: Sexual conflict in a cannibalistic spider. Behav. Ecol. 16, 377–382 (2005).

[b20] Neumann R. & Schneider J. M. Differential investment and size-related mating strategies facilitate extreme size variation in contesting male spiders. Anim. Behav. 101, 107–115 (2015).

[b21] Schneider J. M. & Elgar M. A. Sexual cannibalism and sperm competition in the golden orb-web spider *Nephila plumipes* (Araneoidea): female and male perspectives. Behav. Ecol. 12, 547–552 (2001).

[b22] Schneider J. M. & Michalik P. One-shot genitalia are not an evolutionary dead end - Regained male polygamy in a sperm limited spider species. BMC Evol. Biol. 11, 197 (2011). 21740561 10.1186/1471-2148-11-197 PMC3145602

[b23] Michalik P. & Rittschof C. C. A comparative analysis of the morphology and evolution of permanent sperm depletion in spiders. PLoS One 6, e16014 (2011). 21264312 10.1371/journal.pone.0016014 PMC3019211

[b24] Kuntner M., Zhang S., Gregorič M. & Li D. *Nephila* female gigantism attained through post-maturity molting. J. Arachnol. 40, 345–347 (2012).

[b25] Kuntner M., Gregorič M., Zhang S., Kralj-Fišer S. & Li D. Mating plugs in polyandrous giants: Which sex produces them, when, how and why? PLoS One 7, e40939 (2012). 22829900 10.1371/journal.pone.0040939 PMC3400571

[b26] Ruokolainen L. & Hanski I. Stable coexistence of ecologically identical species: Conspecific aggregation via reproductive interference. J. Anim. Ecol. 85, 638–647 (2016). 26781758 10.1111/1365-2656.12490

